# A comparison of estimated glomerular filtration rates using Cockcroft−Gault and the Chronic Kidney Disease Epidemiology Collaboration estimating equations in HIV infection

**DOI:** 10.1111/hiv.12095

**Published:** 2013-10-03

**Authors:** A Mocroft, L Ryom, P Reiss, H Furrer, A D’Arminio Monforte, J Gatell, S de Wit, M Beniowski, JD Lundgren, O Kirk

**Affiliations:** 1 Department of Infection and Population Health, University College LondonLondon, UK; 2 Copenhagen HIV Program, University of CopenhagenCopenhagen, Denmark; 3 Department of Infectious Diseases, Copenhagen University Hospital, RigshospitaletCopenhagen, Denmark; 4 Academisch Medisch Centrum bij de Universiteit van AmsterdamAmsterdam, The Netherlands; 5 Department of Infectious Diseases, Bern University Hospital and University of BernBern, Switzerland; 6 Infectious and Tropical Diseases Institute, Department of Health Sciences, San Paolo Hospital, University of MilanMilan, Italy; 7 Hospital Clinic i ProvincialBarcelona, Spain; 8 Saint-Pierre HospitalBrussels, Belgium; 9 HIV and AIDS Outpatient Clinic, Szpital SpecjalistycznyChorzow, Poland

**Keywords:** chronic kidney disease, end stage renal disease, eGFR, renal function

## Abstract

**Objectives:**

The aim of this study was to determine whether the Chronic Kidney Disease Epidemiology Collaboration (CKD-EPI)- or Cockcroft−Gault (CG)-based estimated glomerular filtration rates (eGFRs) performs better in the cohort setting for predicting moderate/advanced chronic kidney disease (CKD) or end-stage renal disease (ESRD).

**Methods:**

A total of 9521 persons in the EuroSIDA study contributed 133 873 eGFRs. Poisson regression was used to model the incidence of moderate and advanced CKD (confirmed eGFR < 60 and < 30 mL/min/1.73 m^2^, respectively) or ESRD (fatal/nonfatal) using CG and CKD-EPI eGFRs.

**Results:**

Of 133 873 eGFR values, the ratio of CG to CKD-EPI was ≥ 1.1 in 22 092 (16.5%) and the difference between them (CG minus CKD-EPI) was ≥ 10 mL/min/1.73 m^2^ in 20 867 (15.6%). Differences between CKD-EPI and CG were much greater when CG was not standardized for body surface area (BSA). A total of 403 persons developed moderate CKD using CG [incidence 8.9/1000 person-years of follow-up (PYFU); 95% confidence interval (CI) 8.0–9.8] and 364 using CKD-EPI (incidence 7.3/1000 PYFU; 95% CI 6.5–8.0). CG-derived eGFRs were equal to CKD-EPI-derived eGFRs at predicting ESRD (*n* = 36) and death (*n* = 565), as measured by the Akaike information criterion. CG-based moderate and advanced CKDs were associated with ESRD [adjusted incidence rate ratio (aIRR) 7.17; 95% CI 2.65–19.36 and aIRR 23.46; 95% CI 8.54–64.48, respectively], as were CKD-EPI-based moderate and advanced CKDs (aIRR 12.41; 95% CI 4.74–32.51 and aIRR 12.44; 95% CI 4.83–32.03, respectively).

**Conclusions:**

Differences between eGFRs using CG adjusted for BSA or CKD-EPI were modest. In the absence of a gold standard, the two formulae predicted clinical outcomes with equal precision and can be used to estimate GFR in HIV-positive persons.

## Introduction

With increasing survival rates in HIV-positive persons, there is an increase in the number of studies focusing on non-AIDS-related conditions, including renal disease and markers of renal function, such as glomerular filtration rate (GFR). Two formulae are commonly used to estimate GFR; the Modification of Diet in Renal Disease (MDRD) and Chronic Kidney Disease Epidemiology Collaboration (CKD-EPI) equations. The CKD-EPI equation was developed by the same group as the MDRD equation, addressing some of the limitations of the original MDRD equation 1,2. A third formula, Cockcroft−Gault (CG), estimates creatinine clearance 3. These are based on serum creatinine measurements, which are subject to considerable intrapersonal variation 4, which increases at higher levels of serum creatinine 5. The CG and MDRD equations have been widely used in HIV-positive persons, even though they were derived in HIV-negative persons with other chronic illnesses, while the CKD-EPI formula was derived in a less selected population 6. MDRD and, more recently, CKD-EPI have tended to be the formulae of choice in the general population, with numerous studies suggesting that CKD-EPI is superior to MDRD and CG 7–10. Similar formula preferences exist for HIV-positive persons, as MDRD and CKD-EPI do not require information on body weight, while the CG formula does not require race.

Comparisons of the different eGFR formulae with a gold standard in HIV-positive persons have, to date, been based on small numbers and focused primarily on normal or mildly impaired renal function 11–14, with contradictory results. A larger study of 200 HIV-positive individuals stable on combination antiretroviral therapy (cART) suggested that CKD-EPI performed better than MDRD 15, while epidemiological studies have mainly focused on a comparison of CKD-EPI with MDRD, in the absence of a gold standard 16,17. Clearly, there is an urgent need for comparing the different formulae to a gold standard to determine which has greatest clinical relevance. Although less clinically relevant, it is also relevant to compare formulae within cohort studies, where no gold standard is available, to allow easier cross-comparison of studies and interpretation of results.

The aims of these analyses were therefore to investigate differences between the two most commonly used formulae, CG and CKD-EPI, and to explore which best predicts different levels of chronic kidney disease (CKD), end-stage renal disease (ESRD) or deaths from kidney disease and all-cause mortality.

## Persons

The EuroSIDA study is a prospective, observational cohort study of 18 722 HIV-positive persons in 108 centres across 33 European countries, plus Israel and Argentina. The study has been described in detail previously 18. In brief, persons were enrolled in nine cohorts from May 1994 onwards. Information is collected on a standardized data collection form every 6 months, including all CD4 counts and viral loads measured since the last follow-up and starting and stopping dates of all antiretrovirals. Dates of diagnosis of all clinical AIDS-defining illnesses are recorded using the 1993 clinical definition of AIDS from the Centers for Disease Control and Prevention 19, as well as all deaths, with cause of death determined by the Coding Causes of Death in HIV (CoDe) protocol and by applying a standardized algorithm 20,21. Cardiovascular disease, non-AIDS-defining malignancies, end-stage hepatic encephalopathy, pancreatitis, and ESRD were included as non-AIDS-defining events, as previously described 22. All serum creatinine measurements obtained during routine care have been collected since 1 January 2004.

To ensure correct person selection and to verify that accurate data are supplied, members of the coordinating office visit all centres to check the information provided against case-notes for all persons with clinical events and a randomly selected 10% of persons per year.

## Statistical methods

Creatinine clearance (referred to as eGFR) was calculated using CG and GFR using the CKD-EPI formula 2. Evidence on whether to adjust for body surface area (BSA) when using CG is contradictory 23,24, and our main analyses adjust for BSA 25, with some results presented unadjusted. Weights were required to be measured within 1 year of the serum creatinine measurement for CG eGFR to be calculated. Serum creatinine measurements after 1 January 2004 were included, when routine collection of this data began. The median date of last eGFR was March 2012. A minimum of three eGFRs per person were required. Only eGFRs where the CG was calculated at the same date as CKD-EPI were included to ensure that eGFRs were being compared at similar time-points. Baseline was defined as the first time-point at which eGFR could be calculated with both CG and CKD-EPI. A Bland−Altman plot was used to summarize the differences between the two formulae 26. eGFR groups were defined as normal (≥ 90 mL/min/1.73 m^2^), mildly decreased (60–89 mL/min/1.73 m^2^), moderately decreased (30–59 mL/min/1.73 m^2^) or severely decreased (< 30 mL/min/1.73 m^2^). Models were adjusted for gender, HIV transmission group, ethnic origin, region of Europe, hepatitis B and C status, CD4 count, HIV viral load, combination antiretroviral therapy (cART), prior AIDS or non-AIDS diagnosis, diabetes, hypertension, anaemia, smoking status and cardiovascular disease 22.

Moderate CKD was defined as eGFR <60 mL/min/1.73 m^2^ and advanced CKD as eGFR <30 mL/min/1.73 m^2^ in at least two consecutive measurements at least 3 months apart among patients with an eGFR ≥ 60 mL/min/1.73 m^2^ or ≥ 30 mL/min/1.73 m^2^, respectively, with both formulae. Incidence rates and the proportion progressing to moderate and advanced CKD were calculated for both CG and CKD-EPI, in patients with eGFR ≥ 60 or ≥ 30 at the first eGFR measurement with both formulae. Among those who developed moderate CKD with either formula, logistic regression was used to model the odds of not having moderate CKD with both formulae (discordance), using forwards selection with *P* < 0.1 as entry criterion. Models were adjusted for the same factors as described above. Poisson regression, with time-updated covariates, was used to identify the predictors of moderate CKD using either formula. Patients were followed from baseline until clinical event or last eGFR. Models were adjusted as described above, adjusting additionally for cumulative exposure to antiretrovirals 27. Poisson regression was also used to investigate the relationship between eGFR and ESRD or death from renal disease, or all cause mortality. ESRD was defined as haemodialysis or peritoneal dialysis lasting ≥ 1 month, or a kidney transplant (further information at http://www.cphiv.dk). Renal deaths occurred when renal disease was the underlying cause of death, using the CoDe algorithm 21. Univariate and multivariate models were constructed including current eGFRs, current eGFRs plus moderate and advanced CKD, or moderate and advanced CKD for both clinical endpoints. The fits of the models were compared using the log-likelihood, the Akaike information criterion, and the corrected Akaike information criteria to determine whether CG or CKD-EPI was a better predictor of the clinical endpoints 28.

All statistical analyses were performed using sas version 9.3 (SAS Institute, Cary, NC).

## Results

Of 11 865 persons with at least three eGFRs using CKD-EPI, 9521 (80.2%) also had corresponding eGFR measurements using CG and were included in the analysis. Excluded persons were less likely to be under follow-up in any region compared with Southern Europe, to have hypertension and to be antiretroviral experienced, and more likely to be non-White, to be a former smoker and to have a later baseline date. Characteristics of the 9521 included persons are shown in Table 1. Patients were commonly male (73.7%), White (87.2%) and homosexual (41.0%). At baseline, 29.3% had a prior AIDS diagnosis, 6.5% a prior non-AIDS-related event and 2.9% a prior cardiovascular event. Median CD4 count was 438 cells/μL [interquartile range (IQR) 293–620 cells/μL], median age was 41.9 years (IQR 35.6–49.0 years) and median serum creatinine was 0.89 mg/dL (IQR 0.77–1.00 mg/dL). The number included increased to 10 847 if CG was not standardized for BSA, with similar characteristics to those shown in Table 1.

**Table 1:** Baseline characteristics of 10 487 persons with at least three estimated glomerular filtration rates (eGFRs) using Cockcroft−Gault (CG) and Chronic Kidney Disease Epidemiology Collaboration (CKD-EPI) equations

		*n*	%
All		9521	100
Gender	Male	7013	73.7
	Female	2508	26.3
Race	White	8301	87.2
	Other	1220	12.8
Exposure group	Homosexual	3906	41.0
	IDU	1880	19.7
	Heterosexual	2979	31.3
	Other	756	7.9
Region of Europe	South	2154	22.6
	Central	2645	27.8
	North	2159	22.7
	East	2303	24.2
	Argentina	260	2.7
Hepatitis B	Negative	7691	80.8
	Positive	582	6.1
	Unknown	1248	13.1
Hepatitis C	Negative	5984	62.9
	Positive	2059	21.6
	Unknown	1478	15.5
Prior AIDS diagnosis	Yes	2794	29.3
Prior non-AIDS-related event*	Yes	618	6.5
Prior CV event	Yes	278	2.9
Diabetes	No	8021	84.3
	Yes	461	4.8
	Unknown	1039	10.9
Hypertension	No	5583	58.6
	Yes	2806	29.5
	Unknown	1132	11.9
Smoking	Never	2747	28.8
	Current	3006	31.6
	Former	361	3.8
	Unknown	3407	35.8
Anaemia	No	5080	53.4
	Yes	2018	21.2
	Unknown	2423	25.4
ARV-naïve	Yes	1275	13.4
Ever cART	Yes	8000	84.0
On cART	Yes	7807	82.0
		**Median**	**Interquartile range**
Baseline	(month/year)	01/05	06/04–02/07
CD4 nadir	(cells/μL)	160	60–273
CD4	(cells/μL)	438	293–620
Viral load	(log_10_ copies/mL)	1.69	1.69–3.26
Age	(years)	41.9	35.6–49.0
Serum creatinine	(mg/dL)	0.89	0.77–1.00

ARV, antiretroviral; cART, combination antiretroviral therapy; CV, cardiovascular; IDU, injecting drug use.

Baseline was defined as the first time-point at which eGFR could be calculated with both CG and CKD-EPI.

**Figure 1:**
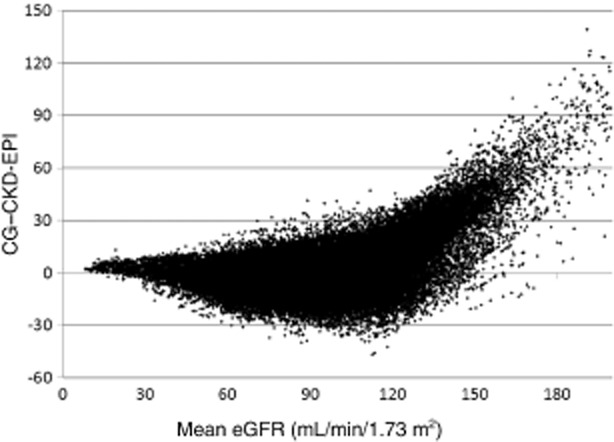
Bland−Altman plot of differences between estimated glomerular filtration rates (eGFRs) calculated using Cockcroft−Gault (CG) and Chronic Kidney Disease Epidemiology Collaboration (CKD-EPI).

There were 133 873 eGFRs with CG standardized for BSA and CKD-EPI, a median of 13 (IQR 7–19) measurements per person and a median time of 3.6 months (IQR 2.8–5.5 months) apart. Of the 133 873 measurements, 114 685 (85.7%) were in agreement; that is, they would be classified as normal, mildly decreased, moderately decreased or severely decreased with both formulae. Figure 1 shows a Bland−Altman plot of the agreement between the two formulae, which varied according to the mean eGFR. On average, the CG eGFR was lower than the CKD-EPI eGFR; the median difference between CKD-EPI and CG (CG minus CKD-EPI) was −0.5 (IQR −6.0 to 5.8) mL/min/1.73 m^2^. In those with severely decreased kidney function, estimated with CG the difference was −2.3 (IQR –4.1 to −0.4) mL/min/1.73 m^2^ and in those with a moderately decreased kidney function it was 3.2 (IQR −0.6 to 7.4) mL/min/1.73 m^2^, compared with 4.2 (IQR −0.6 to 9.0) mL/min/1.73 m^2^ and −2.6 (IQR −10.2 to 3.1) mL/min/1.73 m^2^ in those with mildly decreased or normal kidney function, respectively. Comparing the eGFR groups (normal, mildly decreased, moderately decreased and severely decreased) gave a kappa of 0.75 [95% confidence interval (CI) 0.75–0.76]. The overall correlation coefficient between CG and CKD-EPI (as continuous variables) was 0.885. Correlations and kappas were similar for those on and off antiretroviral therapy.

The agreement between CG and CKD-EPI was considerably lower when CG was not standardized for BSA. Of 143 998 measurements (the higher number reflects the fact that more measurements were available by not standardizing for BSA), 111 747 (77.6%) were in agreement using the categories above. The CG eGFR was generally higher than the CKD-EPI eGFR, with a median overall difference between the CKD-EPI and CG eGFRs (CG minus CKD-EPI) of −5.5 (IQR −18.5 to 5.6) mL/min. There was some variation according to kidney function; in those with severely decreased kidney function, according to CG the difference was −1.9 (IQR –4.8 to −2.5) mL/min and in those with a moderately decreased kidney function it was 7.0 (IQR −0.1 to 14.7 mL/min), compared with 5.3 (IQR −2.9 to 13.7) mL/min and –12.8 (IQR –25.8 to −2.0) mL/min in those with mildly decreased or normal kidney function, respectively. Comparing the eGFR groups (normal, mildly decreased, moderately decreased and severely decreased) gave a kappa of 0.60 (95% CI 0.59–0.60). The overall correlation coefficient between CG and CKD-EPI was also considerably lower at 0.754.

### Moderate and advanced CKD (CG standardized for BSA)

Of 9521 persons, 9121 (95.8%) had an eGFR ≥ 60 mL/min/1.73 m^2^ at baseline with both CG and CKD-EPI, and 9497 (99.7%) had an eGFR ≥ 30 mL/min/1.73 m^2^ at baseline with both formulae (Table 3). The highest incidence of, and progression to, moderate CKD were seen with the CG formula, with an incidence of 8.9 per 1000 person-years of follow-up (PYFU; 95% CI 8.0–9.8), and 4.9% (95% CI 4.4–5.4) estimated to have developed moderate CKD by 6 years after baseline, using Kaplan−Meier estimation. Differences in the incidence or Kaplan−Meier progression rates of advanced CKD between the two formulae were much smaller than those seen for moderate CKD (Table 2).

**Table 2:** Incidence and Kaplan−Meier (KM) estimates of moderate and advanced chronic kidney disease (CKD)

	CG (standardized for BSA)	CKD-EPI
Moderate CKD (*n* = 9121)		
Baseline eGFR [median (IQR)]	98.9 (85.3–113.9)	101.0 (87.9–111.4)
eGFR > 90 mL/min/1.73 m^2^ [*n* (%)]	6056 (66.4)	6505 (71.3)
Events (%)	403 (4.4)	364 (4.0)
PYFU	45 326	50 147
Incidence/1000 PYFU (95% CI)	8.9 (8.0–9.8)	7.3 (6.5–8.0)
KM, 24 months (95% CI)	1.2 (1.0–1.4)	0.9 (0.7–1.1)
KM, 48 months (95% CI)	3.0 (2.6–3.4)	2.4 (2.1–2.7)
KM, 72 months (95% CI)	4.9 (4.4–5.4)	4.0 (3.5–4.5)
Advanced CKD (*n* = 9497)		
Baseline eGFR [median (IQR)]	97.5 (83.7–113.1)	100.2 (86.0–111.0)
eGFR > 90 mL/min/1.73 m^2^ [*n* (%)]	6056 (63.8)	6510 (68.6)
Events (%)	40 (0.4)	45 (0.5)
PYFU	4105	53 055
Incidence/1000 PYFU (95% CI)	0.8 (0.6–1.1)	0.9 (0.6–1.0)
KM, 24 months (95% CI)	0.11 (0.03–0.16)	0.08 (0.02–0.14)
KM, 48 months (95% CI)	0.22 (0.11–0.32)	0.27 (0.16–0.38)
KM, 72 months (95% CI)	0.51 (0.32–0.69)	0.55 (0.37–0.72)

BSA, body surface area; CG, Cockcroft−Gault; CI, confidence interval; CKD-EPI, Chronic Kidney Disease Epidemiology Collaboration; eGFR, estimated glomerular filtration rate; IQR, interquartile range; PYFU, person-years of follow-up.

Moderate CKD was defined as eGFR < 60 mL/min/1.73 m^2^ and advanced CKD as eGFR <30 mL/min/1.73 m^2^ in at least two consecutive measurements at least 3 months apart.

A total of 9121 patients with eGFR > 60/mL/min/1.73 m^3^ at baseline with both formulae were included in estimates of moderate CKD; 9497 patients with eGFR > 30 mL/min/1.73 m^2^ at baseline with both formulae were included in estimates of advanced CKD.

**Table 3:** Odds of discordance [chronic kidney disease (CKD) defined with Cockcroft−Gault (CG) but not Chronic Kidney Disease Epidemiology Collaboration (CKD-EPI) or vice versa] in classification of CKD

		Univariate			Multivariate		
		OR	95% CI	*P*	OR	95% CI	*P*
Ethnicity	White	1.00	–	–	1.00	–	–
	Other	1.63	0.94–2.85	0.085	1.73	0.98–3.06	0.061
HCV antibody	Negative/unknown	1.00	–	–	1.00	–	–
	Positive	0.58	0.36–0.91	0.019	0.56	0.35–0.90	0.016
HIV exposure	MSM/IDU/Het	1.00	–	–	1.00	–	–
	Other	0.49	0.24–0.99	0.046	0.47	0.23–0.97	0.040
Diabetes	No/unknown	1.00	–	–	1.00	–	–
	Yes	0.67	0.38–1.19	0.17	0.63	0.35–1.13	0.12
Baseline date	Per year later	1.23	1.07–1.42	0.0031	1.26	1.09–1.45	0.0020

CI, confidence interval; HCV, hepatitis C virus; Het, heterosexual; IDU, injecting drug user; MSM, men who have sex with men; OR, odds ratio.

A total of 496 persons (5.4%) developed moderate CKD using either CG or CKD-EPI; of these, 271 (54.6%) were classified as having moderate CKD with both formulae (i.e. concordant), 132 with CG but not CKD-EPI (26.6%) and 93 (18.8%) with CKD-EPI but not CG. The 271 persons classified as having CKD with both CKD-EPI and CG formulae were compared with the 225 persons with CKD with either CKD-EPI or CG but not both (Table 3). After adjustment, hepatitis C virus (HCV)-positive persons were less likely to be discordant [adjusted odds ratio (aOR) 0.56; 95% CI 0.35–0.90], as were those with unknown HIV exposure (aOR 0.47; 95% CI 0.23–0.97). Persons of a non-White ethnic origin were more likely be discordant (aOR 1.73; 95% CI 0.98–3.06), as were those with a later baseline date (aOR 1.26 per year later; 95% CI 1.09–1.45). Of 271 who developed CKD with one formula but not the other, 15.1% were HCV-positive, 5.3% were in another HIV exposure group and 14.2% were non-White participants, compared with 23.6, 10.3 and 9.2%, respectively, of the 271 individuals classified as having CKD with both formulae. There were 49 persons (0.5%) who had advanced CKD with CG or CKD-EPI; of these, 36 had advanced CKD with both formulae (73.5%), nine (18.4%) had advanced CKD with CKD-EPI but not CG, and four (8.2%) had advanced CKD with CG and not CKD-EPI. Small numbers precluded any additional analyses.

In general, the same predictors were found for moderate CKD regardless of whether it was defined using CG or CKD-EPI (data not shown). The relationships between cumulative exposure to antiretrovirals and moderate CKD were similar using both the CG and CKD-EPI formulae.

### Predictors of clinical events: comparison of CG (standardized for BSA) and CKD-EPI formulae

**Figure 2:**
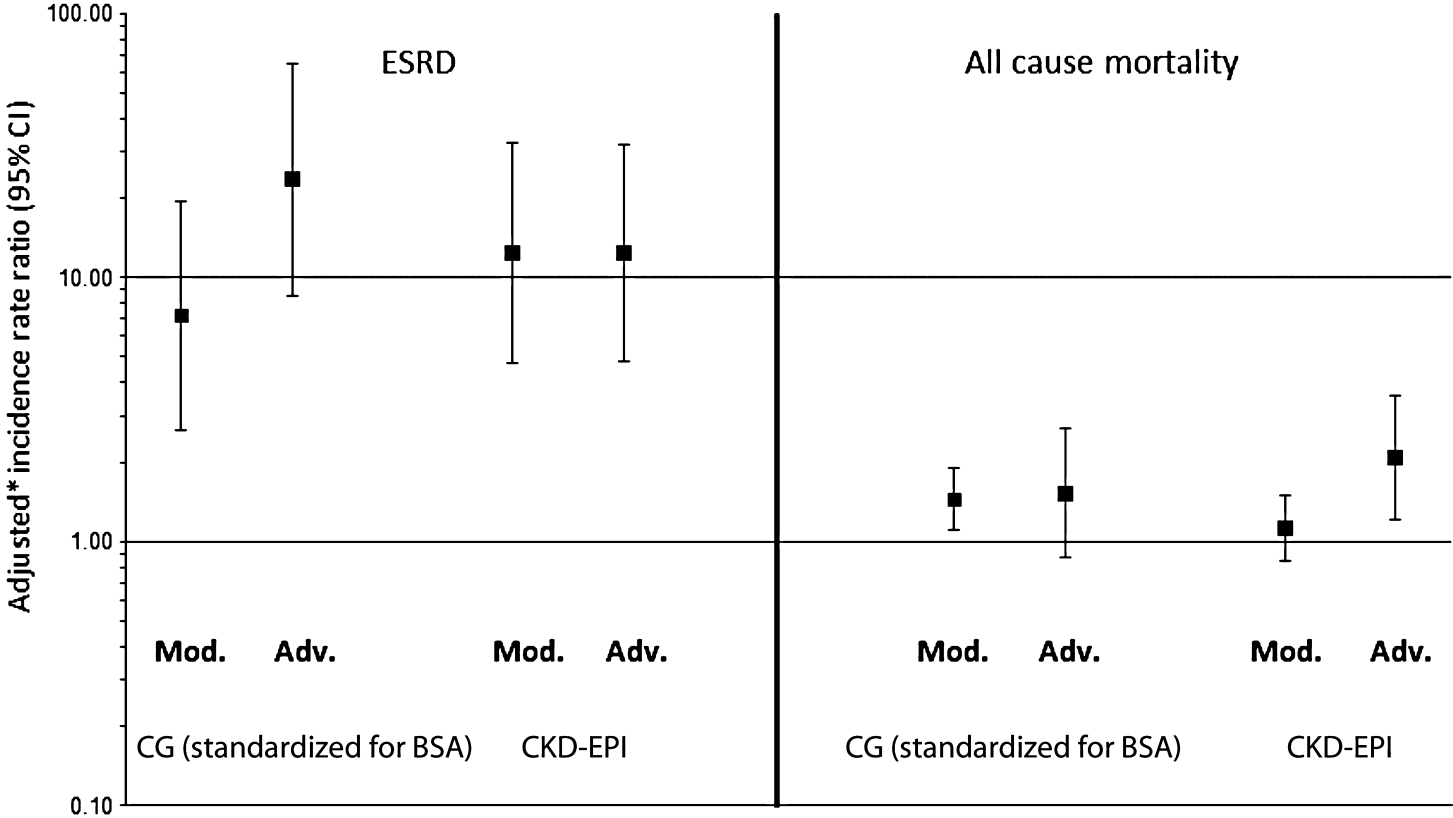
Moderate and advanced chronic kidney disease (CKD) as predictors of fatal and nonfatal end-stage renal disease (ESRD) and mortality. Moderate (Mod.) CKD [confirmed (> 3 months apart) estimated glomerular filtration rate (eGFR) < 60 mL/min/1.73 m^2^] and advanced (Adv.) CKD (confirmed eGFR < 30 mL/min/1.73 m^2^) are included as time-updated variables. *Multivariate models adjusted for gender, race, ethnic origin, region, CD4 count nadir and baseline date as fixed baseline covariates and hepatitis B, hepatitis C, prior AIDS diagnosis, prior non-AIDS-related event (pancreatitis, malignancy and end-stage liver disease for ESRD (and additionally ESRD for mortality), cardiovascular event, diabetes, hypertension, smoking status, anaemia, starting combination antiretroviral therapy, CD4 count, viral load and age as time-updated variables. BSA, body surface area; CG, Cockcroft−Gault; CI, confidence interval; CKD-EPI, Chronic Kidney Disease Epidemiology Collaboration.

There were 36 persons who developed ESRD or who died from renal disease (incidence rate 0.7/1000 PYFU; 95% CI 0.5–0.9) and 565 deaths (incidence 10.8/1000 PYFU; 95% CI 9.9–11.7) during prospective follow-up. CG-derived eGFRs were equal to CKD-EPI-derived eGFRs at predicting both ESRD and death, as measured by a lower Akaike information criterion and log-likelihood, summarized in Figure 2. After adjustment, CG-derived moderate and advanced CKDs were associated with ESRD [adjusted incidence rate ratio (aIRR) 7.17; 95% CI 2.65–19.36 and aIRR 23.46; 95% CI 8.54–64.48, respectively], as were CKD-EPI-derived moderate and advanced CKDs (aIRR 12.41; 95% CI 4.74–32.51 and aIRR 12.44; 95% CI 4.83–32.03, respectively). CG-derived moderate CKD but not advanced CKD was associated with all-cause mortality (aIRR 1.45; 95% CI 1.11–1.90 and aIRR 1.52; 95% CI 0.87–2.67, respectively), while CKD-EPI-derived moderate CKD was not significantly associated with all-cause mortality, but advanced CKD was (aIRR 1.12; 95% CI 0.84–1.50 and aIRR 2.08; 95% CI 1.22–3.57, respectively).

## Discussion

This study of 133 873 eGFRs in 9521 HIV-positive persons showed, in general, modest differences between eGFRs calculated using the CG or CKD-EPI formula. eGFRs were slightly higher with CKD-EPI than with CG. The incidence of moderate CKD was higher using CG than CKD-EPI, although similar HIV- and non-HIV-related risk factors were found, while the incidence of advanced CKD was similar and a rare event with either definition. CG-based eGFRs performed as well as CKD-EPI-based eGFRs in predicting ESRD and all-cause mortality in persons with HIV infection. Our findings are most relevant for epidemiological studies of renal function in HIV-infected persons.

Studies of the general population and HIV-positive persons have typically compared the MDRD and CKD-EPI equations, and found the CKD-EPI equation to be closer to the gold standard 7–10,16,17,29,15. In the absence of a gold standard in this and the majority of epidemiological studies in HIV-infected populations, the predictive ability of the two formulae for key clinical outcomes, such as ESRD or mortality, should be an important consideration. The two formulae predict these clinical outcomes equally from a statistical perspective in this study, but without gold standard GFR measurements, they may be predicting equally well or equally badly. Discordance between CG and CKD-EPI was much higher in our population when CG was not standardized for BSA. All the measures of agreement were considerably lower when CG was not standardized for BSA, including kappa, correlation coefficients and the proportion where the classification of normal, mildly decreased, moderately decreased and severely decreased was the same. In our population, CKD-EPI eGFRs tended to be slightly higher than CG eGFRs when adjusted for BSA, and lower than CKD-EPI eGFRs when not adjusted, suggesting that the population is larger than the standard assumed.

A recent review of non-HIV-positive persons comparing MDRD with CKD-EPI in studies with a reference method suggested that neither MDRD nor CKD-EPI was optimal across all populations and eGFR values 31. The studies comparing CG and CKD-EPI (or MDRD) equations for estimating GFR are primarily confined to highly selected, small, predominantly HIV-negative populations, with conflicting results 12,13,32–34. In HIV-negative persons, less discordance between CG and CKD-EPI or MDRD was found in older persons and those of non-White race 5,35. Some epidemiological studies in HIV infection without access to gold-standard eGFRs may use CG more frequently as the formula does not incorporate race, which may be missing or prohibited for use in analysis in a number of European cohorts, while CKD-EPI may be the formula of choice for cohorts without routine height or weight measurements. Our results show that the formulae are closely correlated and predict CKD and clinical outcomes equally.

It was reassuring that the predictors of moderate CKD were similar regardless of which formula was used, and these predictors have previously been reported in our population 27. In the absence of a gold standard, the incidence of moderate CKD, defined as confirmed eGFR < 60 mL/min/1.73 m^2^ 36, varied depending on the formula used, but was higher using CG compared with CKD-EPI, although the incidences of advanced CKD were similar. This raises issues about person management and resources, and about comparing the incidences of moderate CKD across HIV-positive populations, where there is often considerable heterogeneity in included persons as well as different eGFR formulae used. Ideally, GFR should be directly measured based on the clearance of infused inulin, iohexol, or radiolabelled tracers.

eGFR is measured in the clinical setting for a variety of reasons, including ensuring correct dosage of medications, to monitor changes in eGFR over time, and to assess the risk and development of other complications of progressive renal dysfunction such as bone disease, anaemia and hypertension 37. The use of eGFRs as a surrogate marker for renal function and predictor of clinical events relies on the assumption that changes in eGFRs caused by intervention translate into a change in the risk of ESRD or overall mortality. To date, there is a shortage of data for HIV-positive persons that show that changes in eGFR, however measured, translate into reductions in clinically relevant outcomes 38. We fitted a variety of models, including eGFR values, moderate and advanced CKD, and in all of these the CG eGFR was as good at predicting clinical outcomes as the CKD-EPI eGFR, as measured by the Akaike information criterion.

The limitations of this study should be noted. Our results are most useful for comparisons and interpretation of epidemiological data and not for clinical decision making. Many HIV-infected cohort studies do not routinely collect height or weight, making use of CG impractical, while others are prohibited from collecting information on race, which is included in the CKD-EPI equation. Variation between laboratories in serum creatinine measurements has been well described 5,39, and the CKD-EPI equation requires creatinine measured using enzymatic assays to allow its accurate use. Unfortunately we have no information on the assay used to determine creatinine in our individuals. We have no data on cystatin C, also proposed as a useful measure of kidney function in some studies 40,41, or proteinuria. Finally, it is worth noting that EuroSIDA is a cohort of predominantly White individuals, limiting our power to consider the influence of race, and the number of individuals developing advanced CKD or ESRD was quite low.

In summary, there were modest differences in eGFRs In HIV-positive persons when comparing CG adjusted for BSA and CKD-EPI eGFR formulae. It would be reasonable to use either formula in HIV-positive populations similar to those in EuroSIDA. Although there were no gold-standard GFRs available, predictors of CG- or CKD-EPI-defined moderate CKD were similar and CG eGFRs performed as well as CKD-EPI eGFRs in predicting two key clinical outcomes. In the absence of a large study comparing eGFRs to a gold standard, HIV-infected cohort studies can use either formula, although sensitivity analyses should investigate the robustness of the findings with both formulae, wherever possible.

## Supporting Information

Appendix S1 The EuroSIDA Study Group
