# Supplementary material for: A comparison of estimated glomerular filtration rates using Cockcroft−Gault and the Chronic Kidney Disease Epidemiology Collaboration estimating equations in HIV infection
Source: HIV Med. 2013 Oct 3;15(3):144–52. doi: 10.1111/hiv.12095 (PMC4228765; doi:10.1111/hiv.12095)
Supplement: Appendix S1 — The EuroSIDA Study Group [file hiv0015-0144-sd1.pdf]

Appendix S1. The EuroSIDA Study Group

The multicentre study group on EuroSIDA (national co-ordinators in parentheses).

Argentina: (M. Losso) and M. Kundro, Hospital JM Ramos Mejia, Buenos Aires. Austria: (N. Vetter), Pulmologisches Zentrum der Stadt Wien, Vienna; R. Zangerle, Medical University Innsbruck, Innsbruck. Belarus: (I. Karpov) and A. Vassilenko, Belarus State Medical University, Minsk; V. M. Mitsura, Gomel State Medical University, Gomel; O. Suetnov, Regional AIDS Centre, Svetlogorsk. Belgium: (N. Clumeck), S. De Wit and M. Delforge, Saint-Pierre Hospital, Brussels; R. Colebunders, Institute of Tropical Medicine, Antwerp; L. Vandekerckhove, University Ziekenhuis Gent, Gent. Bosnia-Herzegovina: (V. Hadziosmanovic), Klinicki Centar Univerziteta Sarajevo, Sarajevo. Bulgaria: (K. Kostov), Infectious Diseases Hospital, Sofia. Croatia: (J. Begovac), University Hospital of Infectious Diseases, Zagreb. Czech Republic: (L. Machala) and D. Jilich, Faculty Hospital Bulovka, Prague; D. Sedlacek, Charles University Hospital, Plzen. Denmark: (J. Nielsen), G. Kronborg,T. Benfield and M. Larsen, Hvidovre Hospital, Copenhagen; J. Gerstoft, T. Katzenstein, A-B. E. Hansen and P. Skinhøj, Rigshospitalet, Copenhagen; C. Pedersen, Odense University Hospital, Odense; L. Ostergaard, Skejby Hospital, Aarhus. Estonia: (K. Zilmer), West-Tallinn Central Hospital, Tallinn; J. Smidt, Nakkusosakond Siseklinik, Kohtla-Järve. Finland: (M. Ristola), Helsinki University Central Hospital, Helsinki. France: (C. Katlama), Hôpital de la Pitié-Salpétière, Paris; J-P. Viard, Hôpital Necker-Enfants Malades, Paris; P-M. Girard, Hospital Saint-Antoine, Paris; J. M. Livrozet, Hôpital Edouard Herriot, Lyon; P. Vanhems, University Claude Bernard, Lyon; C. Pradier, Hôpital de l'Archet, Nice; F. Dabis and D. Neau, Unité INSERM, Bordeaux. Germany: (J. Rockstroh), Universitäts Klinik Bonn, Bonn; R. Schmidt, Medizinische Hochschule Hannover, Hannover; J. van Lunzen and O. Degen, University Medical Center Hamburg-Eppendorf, Infectious Diseases Unit, Hamburg; H. J. Stellbrink, IPM Study Center, Hamburg; M. M. Bickel, JW Goethe University Hospital, Frankfurt; J. Bogner, Medizinische Poliklinik, Munich; G. Fätkenheuer, Universität Köln, Cologne. Greece: (J. Kosmidis), P. Gargalianos, G. Xylomenos and J. Perdios, Athens General Hospital, Athens; G. Panos, A. Filandras and E. Karabatsaki, 1st IKA Hospital, Athens; H. Sambatakou, Ippokration General Hospital, Athens. Hungary: (D. Banhegyi), Szent Láslò Hospital, Budapest. Ireland: (F. Mulcahy), St James's Hospital, Dublin. Israel: (I. Yust), D. Turner and M. Burke, Ichilov Hospital, Tel Aviv; S. Pollack and G. Hassoun, Rambam Medical Center, Haifa; S. Maayan, Hadassah University Hospital, Jerusalem. Italy: (S. Vella), Istituto Superiore di Sanità, Rome; R. Esposito, I. Mazeu and C. Mussini, Università Modena, Modena; C. Arici, Ospedale Riuniti, Bergamo; R. Pristera, Ospedale Generale Regionale, Bolzano; F. Mazzotta and A. Gabbuti, Ospedale S Maria Annunziata, Firenze; V. Vullo and M. Lichtner, University di Roma la Sapienza, Rome; A. Chirianni, E. Montesarchio and M. Gargiulo, Presidio Ospedaliero AD Cotugno, Monaldi Hospital, Napoli; G. Antonucci, A. Testa, G. D'Offizi, C. Vlassi, M. Zaccarelli and A. Antorini, Istituto Nazionale Malattie Infettive Lazzaro Spallanzani, Rome; A. Lazzarin, A. Castagna and N. Gianotti, Ospedale San Raffaele, Milan; M. Galli and A. Ridolfo, Osp. L. Sacco, Milan; A. d'Arminio Monforte, Istituto Di Clinica Malattie Infettive e Tropicale, Milan. Latvia: (B. Rozentale) and I. Zeltina, Infectology Centre of Latvia, Riga. Lithuania: (S. Chaplinskas), Lithuanian AIDS Centre, Vilnius. Luxembourg: (T. Staub) and R. Hemmer, Centre Hospitalier, Luxembourg. Netherlands: (P. Reiss), Academisch Medisch Centrum bij de Universiteit van Amsterdam, Amsterdam. Norway: (V. Ormaasen), A. Maeland and J. Bruun, Ullevål Hospital, Oslo. Poland: (B. Knysz) and J. Gasiorowski, Medical University, Wroclaw; A. Horban and E. Bakowska, Centrum Diagnostyki i Terapii AIDS, Warsaw; A. Grzeszczuk and R. Flisiak, Medical University, Bialystok; A. Boron-Kaczmarska, M. Pynka and M. Parczewski, Medical Univesity, Szczecin; M. Beniowski and E. Mularska, Osrodek Diagnostyki i Terapii AIDS, Chorzow; H. Trocha, Medical University, Gdansk; E. Jablonowska, E. Malolepsza and K. Wojcik, Wojewodzki Szpital Specjalistyczny, Lodz. Portugal: (F. Antunes), M. Doroana and L. Caldeira, Hospital Santa Maria, Lisbon; K. Mansinho, Hospital de Egas Moniz, Lisbon; F. Maltez, Hospital Curry Cabral, Lisbon. Romania: (D. Duiculescu), Spitalul de Boli Infectioase si Tropicale: Dr. Victor Babes, Bucarest. Russia: (A. Rakhmanova), Medical Academy Botkin Hospital, St Petersburg; N. Zakharova, St Petersburg AIDS Centre, St Petersburg; S. Buzunova, Novgorod Centre for AIDS, Novgorod. Serbia: (D. Jevtovic), The Institute for Infectious and Tropical Diseases, Belgrade. Slovakia: (M. Mokráš) and D. Staneková, Dérer Hospital, Bratislava. Slovenia: (J. Tomazic), University Clinical Centre Ljubljana, Ljubljana. Spain: (J. González-Lahoz), V. Soriano, P. Labarga and J. Medrano, Hospital Carlos III, Madrid; S. Moreno and J. M. Rodriguez, Hospital Ramon y Cajal, Madrid; B. Clotet, A. Jou, R. Paredes, C. Tural, J. Puig and I. Bravo, Hospital Germans Trias i Pujol, Badalona; J. M. Gatell and J. M. Miró, Hospital Clinic i Provincial, Barcelona; P. Domingo, M. Gutierrez, G. Mateo and M. A. Sambeat, Hospital Sant Pau, Barcelona. Sweden: (A. Blaxhult), Venhaelsan-Sodersjukhuset, Stockholm; L. Flamholc, Malmö University Hospital, Malmö; A. Thalme and A. Sonnerborg, Karolinska University Hospital, Stockholm. Switzerland: (B. Ledergerber) and R. Weber, University Hospital, Zürich; P. Francioli and M. Cavassini, Centre Hospitalier Universitaire Vaudois, Lausanne; B. Hirschel and E. Boffi, Hospital Cantonal Universitaire de

Geneve, Geneve; H. Furrer, Inselspital Bern, Bern; M. Battegay and L. Elzi, University Hospital Basel. Ukraine: (E. Kravchenko) and N. Chentsova, Kiev Centre for AIDS, Kiev; V. Frolov and G. Kutsyna, Luhansk State Medical University; Luhansk; S. Servitskiy, Odessa Region AIDS Center, Odessa; M. Krasnov, Kharkov State Medical University, Kharkov. UK: (S. Barton), St Stephen's Clinic, Chelsea and Westminster Hospital, London; A. M. Johnson and D. Mercey, Royal Free and University College London Medical School, London (University College Campus); A. Phillips, M. A. Johnson and A. Mocroft, Royal Free and University College Medical School, London (Royal Free Campus); M. Murphy, Medical College of Saint Bartholomew's Hospital, London; J. Weber and G. Scullard, Imperial College School of Medicine at St Mary's, London; M. Fisher, Royal Sussex County Hospital, Brighton; C. Leen, Western General Hospital, Edinburgh.

Steering committee: J. Gatell, B. Gazzard, A. Horban, I. Karpov, B. Ledergerber, M. Losso, A. D'Arminio Monforte, C. Pedersen, A. Rakhmanova, M. Ristola, J. Rockstroh (Chair) and S. De Wit (Vice-Chair).

Additional voting members: J. Lundgren, A. Phillips and P. Reiss.

Coordinating centre staff: O. Kirk, A. Mocroft, A. Cozzi-Lepri, D. Grint, A. Schultze, L. Shepherd, M. Sabin, D. Podlekareva, J. Kjær, L. Peters, J. Nielsen, J. Tverland and A. H. Fischer.

EuroSIDA representatives to EuroCoord: O. Kirk, A. Mocroft, J. Grarup, P. Reiss, A. Cozzi-Lepri, R. Thiebaut, J. Rockstroh, D. Burger, R. Paredes, J. Kjær and L. Peters.
